# Identification of formation mechanism and key elements of quality geriatric care behavior of nursing assistants in nursing homes: a grounded theory study

**DOI:** 10.3389/fpubh.2024.1425883

**Published:** 2024-06-27

**Authors:** Lulu Liao, Xue Bai, Xiaoxiao He, Lei Tan, Linghua Yang, Huan Long, Shenglan Huang, Xia Li, Ying Han, Xiao Peng, Huijing Chen, Xiufen Yang, Hui Li, Shuang Wang, Yilan Liu

**Affiliations:** ^1^Department of Nursing, Union Hospital, Tongji Medical College, Huazhong University of Science and Technology, Wuhan, Hubei, China; ^2^School of Nursing, Tongji Medical College, Huazhong University of Science and Technology, Wuhan, Hubei, China; ^3^Jianghan District Social Welfare Institute, Wuhan, Hubei, China; ^4^Wuhan Social Welfare Institute, Wuhan, Hubei, China; ^5^Changsha First Social Welfare Institute, Changsha, Hunan, China; ^6^Yueyang City Social Welfare Institute, Yueyang, Hunan, China; ^7^Xinyang City Shengde Nursing Home, Xinyang, Henan, China; ^8^Xianning Central Hospital, Xianning, Hubei, China; ^9^Health Science Center, Yangtze University, Jingzhou, Hubei, China; ^10^Personal Pension Business Department, Head Office, Ping An Pension Insurance, Shanghai, China; ^11^Department of Geriatric, Shenzhen People’s Hospital, Shenzhen, Guangdong, China; ^12^Department of Breast and Nail Surgery, Third Xiangya Hospital, Central South University, Changsha, Hunan, China

**Keywords:** quality care behavior, nursing assistants, nursing homes, formation mechanism, grounded theory

## Abstract

**Objective(s):**

This study aimed to identify the key elements and develop a formation mechanism model of quality geriatric care behavior for nursing assistants.

**Methods:**

This qualitative research employed the strategy of grounded theory proposed by Strauss and Corbin. Furthermore, the data was collected by participatory observation and semi-structured interviews. A total of 12 nursing managers, 63 nursing assistants, and 36 older people from 9 nursing homes in 6 cities were interviewed, whereas for the observatory survey, participants were recruited from 2 nursing homes.

**Results:**

The comparative and analysis process revealed 5 key elements of quality geriatric care behavior, including holistic care, personalized care, respect, positive interaction, and empowerment. Based on the Capability-Opportunity-Motivation-Behavior (COM-B) model, key elements and the 3 stages of quality geriatric care behavior (negative behavior cognition stage, practice exchange run-in stage, and positive behavior reinforcement stage), the theoretical framework of the formation mechanism was established.

**Conclusion:**

The results indicated that nursing assistants’ capabilities, motivation, and organizational and environmental support are vital for quality care behaviors. The theoretical framework established in this study provides theoretical support and practical reference to policymakers, institutional administrators, and healthcare professionals for improving nursing assistant’s care behaviors.

## 1 Introduction

With the increasing age of the Chinese population, it has been estimated that by 2030, the aged population will reach 363 million (24.8% of the total population) (1). Furthermore, the number of incapacitated older adult will increase from 43.75 million in 2020 to 91.4 million in 2050 (2). Currently, about 15 million older adult people have dementia in China (3), and the number of incapacitated, semi-incapacitated, and dementia older adult people is steadily rising, which has increased the demand for old-age care and medical services (4). Compared with the traditional family and community pension model, institutional pension service personnel have a relatively higher level of specialization with more efficient facilities and equipment, which is the preferred pension model for the older adult with special needs (5). However, these characteristics increase the vulnerability of older people, increasing their risk of abuse or neglect in facilities (6). Moreover, because of the negative media reports (the cases of older adult abuse), older adult people and their families are reluctant for institutional care (7). When the basic institutional resident’s needs are fulfilled, their concern for a higher quality of life becomes preeminent.

Currently, the institutional older adult care services in China are under extensive quantity development, and there is insufficient attention to service quality (8). A multicenter study analyzed 35 nursing homes in China and indicated that only 57.9% of nursing homes have an Observable Indicators of Nursing Home Care Quality (OIQ) score > 103, indicating they reached the required quality; however, certain work is still required to achieve high quality (9). The core factor that allows residents of nursing homes to get quality care services is the nursing assistants (10). Nursing assistants, as the frontline major force, are frequently in contact with the residents; therefore, improving their care behavior is crucial for enhancing the quality of older adult care services (11). Recently, a systematic review of older adult abuse incidence in nursing homes indicated that 64.2% of abuse, predominantly psychological abuse and neglect, was caused by the nursing staff (12). The Chinese nursing assistants are generally older, and their cultural background is low (13). Furthermore, some of these assistants lack basic courtesy and respect and might be unkind and rude, which damages the dignity of the older adult (14) and may also affect their trust in the care service. A survey of 1,544 staff across 92 English care home units revealed that very few staff carried out specific person-centered activities (15). Moreover, a qualitative study indicated that nursing assistants’ caring behavior needs further improvement to meet the diverse needs of residents care (16). Therefore, nursing assistants have geriatric care behavioral problems that require improvements.

Some studies define quality care as aging-sensitive, evidence-based, individualized care that promotes informed decision-making and is continuous across settings (17, 18). In addition, promoting independence and autonomy was also argued to be crucial for quality care to the older adult (19). It has been suggested that personal factors (burnout and stereotyping of the older adult) and work-related factors (management support) were associated with good care in nursing homes (20). A survey of 2,898 nursing staff in nursing homes showed that the working time of nursing staff is associated with high-quality care behavior, and 3–5 years of experience has the strongest correlation with high-quality indicators (21). Most existing studies are focused on the identification and verification of quality geriatric care behavior’s influencing factors; however, comprehensive investigations on the formation mechanism and geriatric care behavior’s after-effects are still lacking (22–24). Therefore, this study aimed to examine the key elements and formation process of quality geriatric care behavior for nursing assistants. Furthermore, this process also benefits from the COM-B model, which can provide an appropriate framework for explaining how quality geriatric care behavior occurs. The data of this investigation will provide information for institutional administrators, geriatric nurse educators, and other professionals on how to support nursing assistants in improving their care behavior.

## 2 Methods

### 2.1 Design

**Figure 1:**
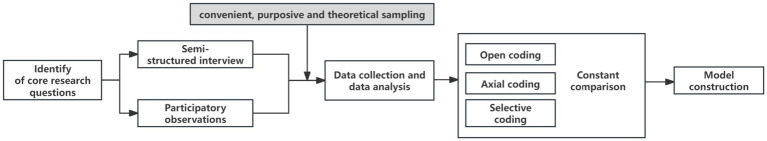
The research process.

This study employed the strategy of grounded theory proposed by Strauss and Corbin to explore the key elements and formation process of quality geriatric care behavior for nursing assistants. Grounded theory is a qualitative research method based on symbolic interactionism (25) and the philosophy of pragmatism (26). The symbolic interactionism assumes that interactions between people are constantly changing and interpretive. Furthermore, it focuses on how individuals create, interpret, approve, and modify the meanings and actions in their daily lives (27). Figure 1 indicates the research process employed. Moreover, the good reporting of a CASP Qualitative checklist was observed for this article (see Supplementary File 1).

The grounded theory provides flexible recommendations for the process as it allows the identification of information and the acquisition of themes and theories based on the majority’s perspectives (28, 29). Researchers are significantly studying the concepts and theories of social contexts and interactions for sharing different perspectives and views, as well as understanding and interpreting the data (30).

### 2.2 Sampling methods

This study employed convenient, purposive, and theoretical sampling methods. To maximize the identification of concepts related to the research topic, a pilot study was conducted on small, medium, and large nursing homes. However, due to the limited number and professional quality of nursing assistants in these small nursing homes, the acquired information on how to implement quality geriatric care was limited. Therefore, during the formal survey stage, medium and large nursing homes were selected for investigation through convenient sampling.

After determining the nursing homes through convenient sampling, purposive and theoretical samplings were used to select participants for the interview. In the early stages, researchers selected individuals who agreed to the interview for a maximum diversity sample based on age, years of work, marital status, etc. Moreover, the theoretical sampling method was based on simultaneous data collection and analysis. Researchers synchronized the data collection and analysis, using theoretical sampling methods to select the next interviewee based on gradually formed conceptual categories until the data reached saturation (31).

### 2.3 Participants

The interviewees were nurse managers, nursing assistants, and older people from 9 nursing homes located in central regions of China, including 2 in Wuhan (Hubei), 1 in Xianning (Hubei), 2 in Changsha (Hunan), 1 in Yueyang (Hunan), 2 in Zhengzhou (Henan), and 1 in Xinyang (Henan). The face-to-face, semi-structured interviews were conducted from July to October 2023.

The researchers selected 9 nursing homes in central China (Hubei, Hunan, and Henan) according to accessibility and convenience. Eligibility criteria for nursing home selection included older adult care institutions that (a) have obtained the establishment license issued by the Department of Civil Affairs and are in normal operation, (b) have more than 100 beds and 80 or more permanent residents, (c) have more than 40 nursing assistants, and (d) were willing to participate in the study.

For data collection, the researchers informed the managers about the study and asked for assistance in the selection of appropriate nursing assistants and older people as the interviewees based on the nursing home conditions. The nursing managers: (a) those who were in medium and large older adult care institutions, (b) those with >3 years of working experience in old-age care quality management, (c) those with an honored college diploma and above, (d) ≥ 18 years of age, and (e) who voluntary agreement to participate in this study were included. The inclusion criteria for nursing assistants included nursing assistants (a) who were in medium and large older adult care institutions, (b) who held a national vocational qualification for nursing assistants, (c) who had >1 year experience in older adult care service, (d) were ≥ 18 years of age and (e) who voluntarily joined the study and provided signed informed consent. The inclusion criteria for older people were as follows: (a) non-terminal older adult aged 60 years and above, (b) who were living in the nursing homes for >3 months, (c) had no history of mental illness, (d) could understand and communicate without barriers and (e) voluntary agreed to participate in this study.

### 2.4 Instrument

Interviews were conducted using a semi-structured interview guide developed through discussions with the research team. The instrument comprised the informed consent form, general background information of respondents, and the interview outline (Table 1).

**Table 1:** Interview questions and the corresponding interviewees.

Interviewees	Interview questions
Nursing managers	Please speak about your feelings and understanding of quality geriatric care. What characteristics would you attribute to a nursing assistant who provides quality geriatric care to the residents? What negative behaviors do you know about nursing assistants? And how to solve it? What factors do you think impact the implementation of quality geriatric care for nursing assistants? How do you think to improve quality geriatric care behavior for nursing assistants? What influence do you think the quality geriatric care behavior of nursing assistants has?
Nursing assistants	How do you care for the older adult? How do you feel about your working performance? Can you tell me something impressive about your job? How do you determine which actions are positive and which are negative? What did you do when you realized that the behavior was wrong? What qualities or characteristics do you think a good nursing assistant should have? What factors do you think affect your caregiving behavior? How do you think you can improve your caregiving behavior? What value or impact do you believe about improving your caregiving behavior?
Older people	Why did you choose this nursing home? How has your stay affected your life? What do you think of the care provided by nursing assistants? What do you think of what they did? What do you suggest? What factors do you think make the nursing assistants do better or worse?

### 2.5 Data collection and sampling

#### 2.5.1 Semi-structured interview

A pilot interview was conducted in June 2023 to test the interview questions, guides, and forms in a nursing home in Wuhan. Then, the research group discussed and summarized the process of pre-interview and data arrangement and analysis, reflecting and improving the researcher’s errors in the interview, such as the control of the topic, the direction of the interview dialogue, and the follow-up of the interview details. Semi-structured interviews were conducted by one PhD one and Master’s of Nursing researchers (LLL and HXX) between July and October 2023. One-to-one interviews were conducted with the nursing managers and older people, while for the nurse assistants, focus group discussions were performed. All the interviews and focus groups were held in meeting rooms at the nursing homes, except for one-to-one interviews with the older adult, which were conducted in their single room. The focus group discussion and interviews lasted for 40–90 min. All the interviews and discussions were audio recorded and then transcribed verbatim within 48 h of each interview. In addition, the first author wrote field notes to record non-verbal information during the interviews and focus groups. The results of each interview were summarized by the researcher and confirmed by the participant.

#### 2.5.2 Participatory observations

The researchers contacted a nursing home in Yuhua District (Changsha City, Hunan Province) and one in Jianghan District (Wuhan City, Hubei Province) to inform their Heads of the purpose and observations of this study and obtained their consent and assistance. The researcher entered the institution as a trainee, stayed in each institution for 2 weeks, and closely observed the nursing behavior of the older adult care workers, focusing on the interaction between the nursing workers and the older adult, the words and deeds of the nursing assistants, and the caregiving behavior. The observation topic was consistent with the topic outlined in the interview. After each observation, the researcher wrote descriptive and reflective records, as well as field observations.

### 2.6 Data analysis

The participant’s basic characteristics are presented by constituent ratios or mean and standard deviation (SD). Data for this analysis was predominantly coded by LLL, with assistance from HXX, using NVivo12.0. This software assists in the preliminary open coding and conceptualization, as well as the unified data management. The later analysis of categories, attributes, dimensions, and their relationships was carried out using Word files. The visualization function of NVivo 12.0 was employed for visualization and data relationship analysis. The collection, collation, and analysis of data were carried out dynamically and synchronously. Immediately after data collection, the collation and analysis were initiated, and the data collection was stopped when no new code appeared. Data saturation was determined by the research team (32). In this study, constant comparative process and three-level coding (open, axial, and selective codings) were used for data analysis (33, 34).

Open coding is the first step in analysis and involves dividing the transcript into segments. After continuous comparison and brainstorming, the researchers selected a topic and assigned the initial code or tags to the data, thereby categorizing large scattered and mixed data into different categories. The second step of Grounded Theory is axial coding, which primarily discusses the concept of first-level coding, represents the relationship between them, and forms second-level categories. The last step is selective coding, which is the process of further integrating and condensing analysis results, finding core categories, as well as forming storylines and frameworks. The researchers further evaluated second-level categories and the relationship between them that emerged during the axial coding phase.

The grounded theory emphasizes the concept of “theoretical saturation”. As data are collected and theories are developed, a point is reached where additional data is not significant for interpretation (28). In this study, the theoretical saturation test was conducted by analyzing the remaining 25% of the transcript using the acquired first and second-level categories. During data analysis, the research team regularly discussed data interpretation and conceptualization. Theoretical saturation was reached after conducting interviews at the 8th nursing home and confirmed after the 9th interview.

### 2.7 Study rigor

To ensure the reliability of data, credibility, transferability, dependability, conformability, and authenticity were considered (35). Credibility was achieved by audiotaping the interviews and taking detailed field notes during participant observation. The findings were discussed by 5 aged care specialists who formed an auditing panel. This multicenter study validated the results by citing interview excerpts and seeking evidence. Furthermore, to improve the transferability of the study, in-depth research was conducted on the social background of Chinese nursing homes. To ensure the dependability of the study, data were transcribed, and the transcripts and interview summaries were returned to the participants for verification and addition. The study’s conformability was enhanced by linking the researchers’ explanations with the participants’ quotes. Finally, the authenticity was established by the verbatim transcripts and field notes.

### 2.8 Ethics approval and consent to participate

This research was reviewed and approved by the Ethics Committee of the College (NO. 2023-S098). The study followed the guidelines and regulations of the Helsinki Declaration. All the participants were explained about the study before the interviews, and their signed written informed consent were acquired. Numbers were used instead of names to protect the participants’ confidentiality. And Written informed consent was obtained from the [individual(s) AND/OR minor(s)’ legal guardian/next of kin] for the publication of any potentially identifiable images or data included in this article.

## 3 Results

### 3.1 Demographics

In total, 111 participants were surveyed in 9 nursing homes in 6 cities, including 12 nursing managers (mean age = 43.8), 63 nursing assistants (mean age = 46.2), and 36 older people (mean age = 81.6). Table 2 presents the detailed demographic information of the participants. The personal information of participants was removed, and every interviewee was given a unique code. The 12 nursing managers, 63 nursing assistants, and 36 older people were identified by the codes as M1–12, N1–63, and P1–36, respectively.

**Table 2:** The demographic data of participants (*N* = 111).

Participants’ characteristics	Nursing managers (*N* = 12)	Nursing assistants (*N* = 63)	Older people (*N* = 36)
Age^a^	43.8 (35.8–50.5)	46.2 (39.0–53.0)	81.6 (78.0–87.0)
Years of work^a^	8.6 (3.3–10.0)^b^	7.8 (3.0–11.0)	None
Gender^c^			
Male	0 (0.0)	3 (4.8)	5 (13.9)
Female	12 (100.0)	60 (95.2)	31 (86.1)
Ethnicity^c^			
Han ethnicity	12 (100.0)	59 (93.7)	36 (100.0)
Tujia ethnic minorities	0 (0.0)	3 (4.7)	0 (0.0)
Hmong ethnic minorities	0 (0.0)	1 (1.6)	0 (0.0)
Education levels^c^			
Elementary	0 (0.0)	8 (12.7)	7 (19.4)
Junior high	0 (0.0)	39 (61.9)	9 (25.0)
Senior high	0 (0.0)	3 (4.8)	12 (33.3)
Post-secondary	4 (33.3)	9 (14.3)	6 (16.7)
Undergraduate	8 (66.7)	4 (6.3)	2 (5.6)
Marital status^c^			
Single	0 (0.0)	6 (9.5)	0 (0.0)
Married	11 (91.7)	57 (90.5)	24 (66.7)
Divorced/separated	1 (8.3)	0 (0.0)	0 (0.0)
Widowed	0 (0.0)	0 (0.0)	12 (33.3)
Position^c^			
Junior care assistant	0 (0.0)	34 (54.0)	None
Mediate care assistant	0 (0.0)	17 (27.0)	None
Senior care assistant	0 (0.0)	12 (19.0)	None
Nurse	3 (25.0)	0 (0.0)	None
Senior nurse	3 (25.0)	0 (0.0)	None
Supervisor nurse	3 (25.0)	0 (0.0)	None
Deputy chief nurse	0 (0.0)	0 (0.0)	None
Chief nurse	3 (25.0)	0 (0.0)	None

a Median (IQR).

b Years of managerial work for managers.

c *N* (%).

### 3.2 Coding analysis results

**Figure 2:**
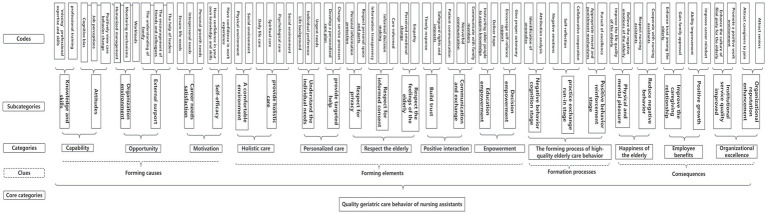
The theoretical structure diagram of the quality geriatric care behavior formation mechanism for nursing assistants.

Based on the interview data of 111 respondents and the results of participant’s observation (outcomes in Supplementary File 2), the core category of “quality geriatric care behavior of nursing assistants” was formed via open, axial, and selective coding. A total of 12 categories, 26 subcategories, and 62 codes were formed by repeated comparison, integration, and generalization. The generation process is shown in Figure 2. In the next section, the data is elaborated based on the clues, formation causes, formation elements, formation processes, and consequences.

#### 3.2.1 The formation causes of quality geriatric care behavior

The formation causes of quality geriatric care behavior included nursing assistants’ capability, as well as the opportunity and motivation to implement quality geriatric care behavior.

##### 3.2.1.1 Nursing assistants’ capability

Nursing assistants’ capability regulates the connection between motivation and care behavior, which directly affects care behavior and can be modulated by influencing motivation. Nursing assistants’ capabilities include knowledge, skills, and attitudes.

Knowledge and skills include “missing” professional expertise and skills, as well as professional training. Enhancing the training of nursing assistants can increase their knowledge and standardize their behavior.

*Because there are junior, middle, and senior nursing assistants, you know. Then, the corresponding professional teaching materials, the Ministry of Civil Affairs, have been updated. But other training, such as how to communicate effectively with the older adult, and how to care more humanely, involves very little, mainly for the basic knowledge of daily life care* (M1).

Furthermore, attitudes include cognitive bias, job perceptions, and alterations in positive view-care behavior. Nursing assistants may label the older adult as incompetent and dependent. Positive job perceptions refer to the positive cognition and feelings of nursing assistants towards their occupation, including professional image and career identity.

*I feel that I am quite suitable for this job; I also like it, especially chatting with old people. I can learn a lot; their life experience will give me some help* (N20).

##### 3.2.1.2 Opportunity to implement quality geriatric care behavior

The opportunity to implement quality geriatric care behavior can directly affect care behavior, which is also affected by influencing motivation. The opportunity to implement quality geriatric care behavior includes organizational environment and external support. The organizational environment includes humanized management, monitoring mechanisms, and workloads.

*Our facility pays great attention to the mental health of the nursing assistants; for example, every October, we will hold Nursing Staff Day, and our institution has a special sand table activity room for nursing staff to decompress; if they are not happy, you cannot expect them to take good care of the older adult. It would be possible to take out their anger on the old people…* (M10).

The external support includes family understanding, encouragement from friends and colleagues, and the leader’s help.

*A word of encouragement from a colleague, assistance from a leader, or even a bit of support from family can greatly boost me. Last week, an older adult person was very agitated due to insomnia. I tried my best to comfort her, but it was not very effective. My colleague suggested using some soft music and deep breathing techniques. His support and suggestions not only helped the older adult person but also taught me a new skill* (N21).

##### 3.2.1.3 Motivation to implement quality geriatric care behavior

Motivation is a component of the COM-B model, and quality care behavior requires strong motivation. The motivation for implementing quality geriatric care behavior includes career needs, satisfaction, and self-efficacy. An individual is motivated when a need is activated and requires satisfaction, which drives behavior. Career needs satisfaction includes ensured life, interpersonal fulfillment, and personal growth.

*I have a monthly salary of more than 3,000, nearly 4,000; here they (institutions) also bought us five insurances and one fund. As long as I do well here, and get my senior certificate, I can continue to work in a private care facility when I retire. If you do not do well, often by the older adult or family complaints, or deducted wages, more times, maybe dismissed…* (N2).

Self-efficacy is an important factor that affects quality care behavior and includes confidence in one’s knowledge and skills as well as work experience.

*Due to the lack of work experience, they are also easily influenced by their colleagues and blindly follow their colleagues’ bad behavior without realizing it. Therefore, we will adopt green hands under the supervision of seniors so that new employees will be fixed with an experienced nursing assistant* (M9).

#### 3.2.2 The formation elements of quality geriatric care behavior

The formation elements included holistic care, personalized care, older adult respect, positive interaction, and empowerment. Holistic care is defined as the process of caring for the older adult. Therefore, nursing assistants should build a comfortable environment for the older adult and provide holistic care.

*I think nursing assistants should not only play the role of caregiver but also play the role of family members and relatives, not only to provide daily life photos, such as psychological counseling and safe behavior education, which should be noted that clothing, food, housing, and activities are only the most basic* (M10).

Individual care is defined as the process of care. The nursing assistants should understand the individual needs of the older adult and provide targeted help.

*Is the food soft or hard? The living habits of the older adult should be understood clearly, which is what we often say to provide personalized care. The older adult people have different ethnic beliefs and eat different food. If there are Hui older adult people in our institution, they have to provide special meals* (N9).

When caring for the older adult, their privacy should be respected, their right to informed consent should be practiced, and their feelings should be considered.

*When a nursing assistant was scrubbing a bedridden older adult woman, she closed the bed curtains even though the woman was completely immobile (and suffering from severe dementia)* (Participatory observation records).

Positive interaction includes building trust, communication, and exchange.

*They (Nursing assistants) should listen to our voices more; once, I was in a bad mood, but she did not notice it, making me even unhappy* (P27).

*Although the bedridden older adult woman suffered from cognitive impairment and was unable to communicate normally, the nurse kept saying to her while scrubbing her body, “It will make you more comfortable after washing…”* (Participatory observation records).

For nursing assistants, empowerment means providing them with information channels, allowing knowledge gain, and encouraging them to participate in decision-making.

*You want them to gain some ‘autonomy’ in this cared-for environment. The menu is different every day, so they can choose according to their taste and make suggestions* (N38).

#### 3.2.3 The formation processes of quality geriatric care behavior

The formation processes of quality geriatric care behavior include the negative behavior cognition stage, practice exchange run-in stage, and positive behavior reinforcement stage. Negative behavior cognition is the initial stage, which mainly comprises 2 sub-stages: identification of anomalies and attribution analysis.

*Last time, I told another nursing assistant that is when taking care of the older adult in bed, you should talk to him more; in fact, he understands, he knows your voice…* (N34).

The practice exchange run-in is the transitional stage with 3 sub-stages: negative emotions, self-reflection, and collaborative cooperation.

*I do not like it when the other staff members constantly judge my behavior; do I have to follow their actions?* (N45).

The positive behavior reinforcement stage includes the establishment of appropriate reward and punishment mechanisms.

*We have a commendation conference every quarter, do a good job, and you will be nominated; I am more and more motivated…* (N51).

#### 3.2.4 The consequences of quality geriatric care behavior

The quality of geriatric care behavior of nursing assistants ultimately increases the well-being of the older adult, employee benefits, and organizational excellence. Furthermore, it significantly affects the older adult, primarily inducing physical and mental pleasure and reducing their negative behavior.

*It will delay their functional decline, and they (older people) will be more satisfied and happy…* (P32).

Moreover, quality geriatric care behavior also markedly influences the nursing assistants, such as improving their caregiving relationships and positive growth.

*I think so, if the work is done well, the older adult and their families will be satisfied, and our work will be more satisfactory. In this process, I will also gain a lot* (P37).

Organizational excellence refers to the increased quality of care services, reduced organizational reputation improvement, and the ultimate realization of organizational change and development.

*Moreover, family members will also help us publicize for free, saying that our facility is good, the older adult will always live here, and the occupancy rate has also risen* (M7).

### 3.3 Theoretical framework of the formation mechanism of quality geriatric care behavior

**Figure 3:**
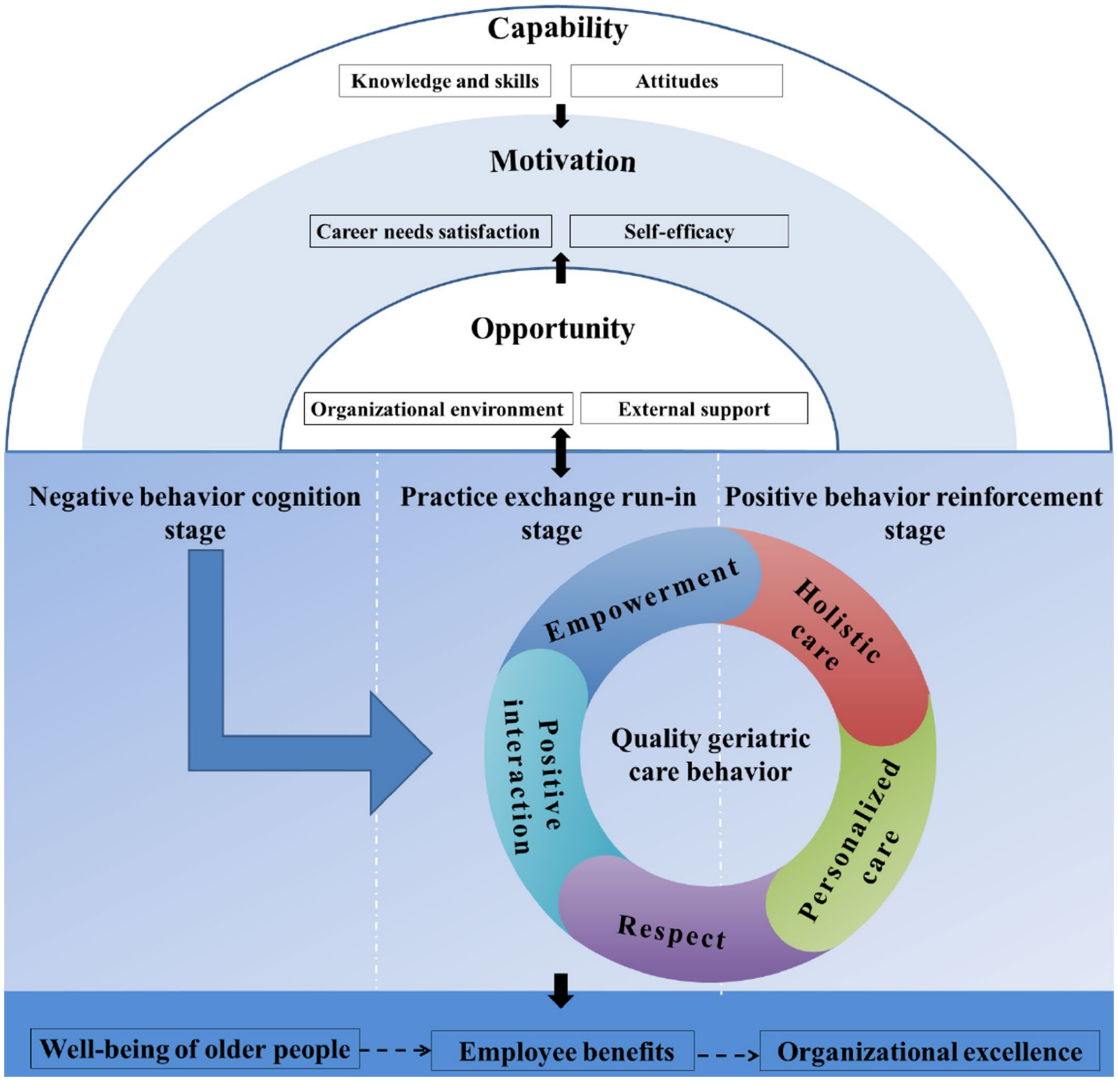
The theoretical framework of the quality geriatric care behavior formation mechanism for nursing assistants.

Based on the above coding analysis results of continuous analysis, comparison, integration of the inter-category relationship, and the story clues, the mechanism theoretical framework of quality geriatric care behavior of nursing assistants was established (Figure 3). Furthermore, during analysis, the researchers correlated the categories by drawing pictures in the memos and continuously adding these associations in the data analysis, laying the foundation for the theoretical framework. It was revealed that the causes of quality geriatric care behavior formation of nursing assistants were closely related to their ability, motivation, and institutional environment. The COM-B model can systematically describe these factors and their interrelationships; therefore, this model was employed as a partial explanation during the construction of the theoretical framework of quality geriatric care behavior of nursing assistants.

**Figure 4:**
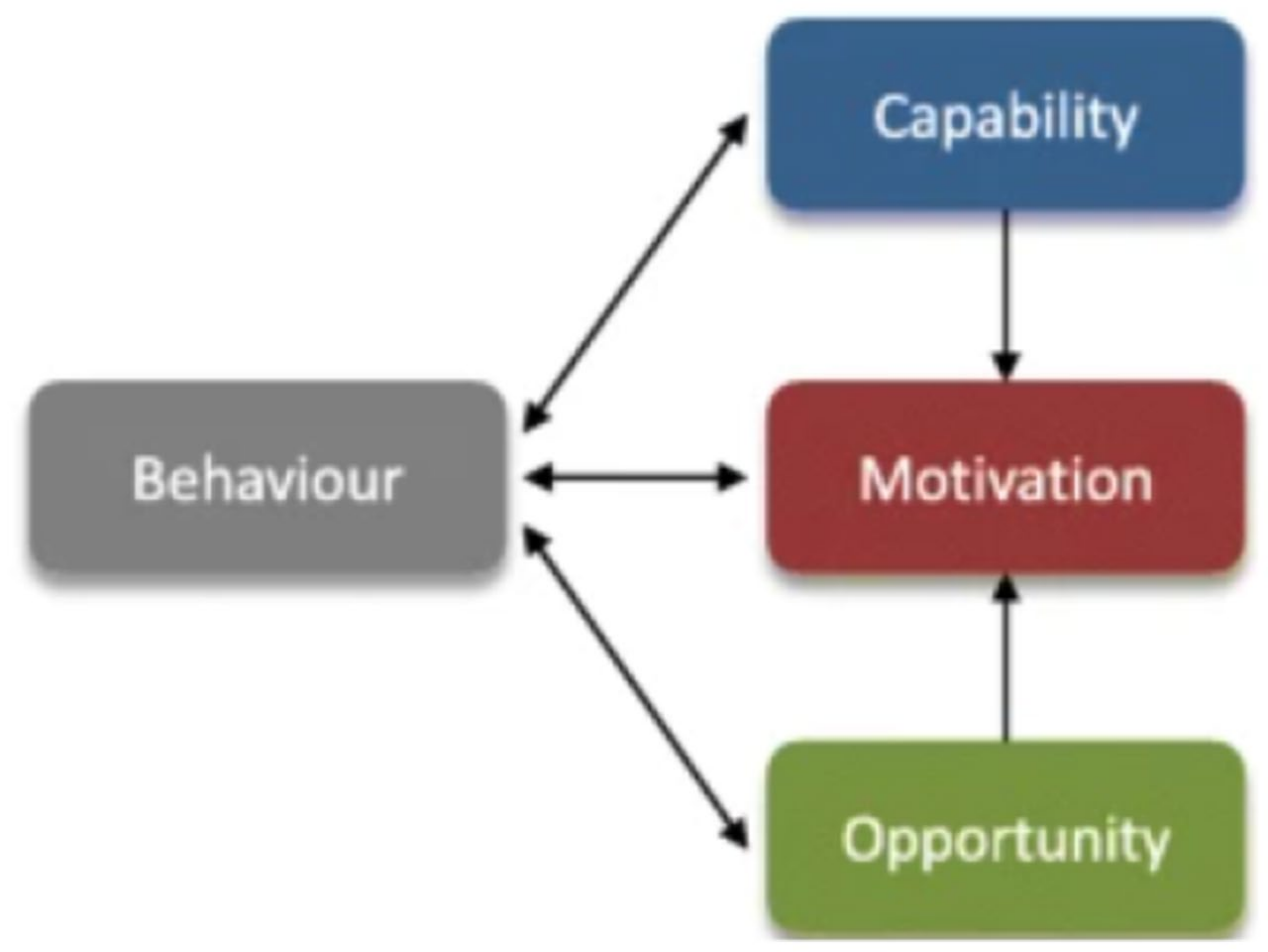
Capability-opportunity-motivation-behavior (COM-B) model.

The causes for the formation of quality geriatric care behavior include the capabilities of nursing assistants, as well as the opportunities and motivations that affect their quality geriatric care behavior. The COM-B model revealed that behavior is a result of an interaction between capability, opportunity, and motivation. Capability and opportunity directly influence motivation, which indirectly affects capability and opportunity through behavior, which in turn influences the behavior (Figure 4) (36). Therefore, in the theoretical framework, the top layer is capability (knowledge, skills, and attitudes), the middle layer is motivation (career needs satisfaction and self-efficacy), and the bottom layer is opportunity (organizational environment and external support). Since the boundaries of the three layer factors are separated, solid lines are used in the innermost and middle layer circles. Furthermore, both capability and opportunity layers have black arrows that point at the motivation layer, indicating that these two factors affect motivation. Moreover, a black two-way arrow is also present between the semicircles and the quality geriatric care behavior process, indicating that there is a dual influence between capability, opportunity, motivation (the elements of the whole semicircle) and behavior.

The key elements of quality geriatric care behavior include holistic care, personalized care, respect for the older adult, positive interaction, and empowerment. These key elements are grouped into a circle that closely revolves around “quality geriatric care behavior.” Moreover, it was observed that the formation and development of quality geriatric care behavior is a dynamic process. In the theoretical framework diagram, the blue arrow direction represents the dynamic development process of the nursing assistants from the negative behavior cognition period to the positive behavior strengthening period. The three stages are separated by dotted lines, where the “quality geriatric care behavior” was only observed in the practice exchange running-in and positive behavior strengthening periods; however, the degree of behavior performance in the two stages was different.

The impact of quality geriatric care behavior of nursing assistants is an important part of the quality geriatric care behavior formation mechanism because every behavior has its cause, process, and consequence. In this study, the impact of nursing assistants’ quality geriatric care behavior was classified into three categories: older adult happiness, employee benefit, and organizational excellence. Where older adult happiness (such as the reduction of negative behavior) affects employee benefit (such as improved care relationships) and organizational excellence (such as improved reputation). These relationships may exist, but not necessarily; therefore, they are represented by a dashed arrow.

Based on the above analysis of the theoretical frameworks, the formation mechanism of quality geriatric care behavior of nursing assistants can be interpreted as the optimized process of their practical experience under professional training and emotional support. Without intervention, nursing assistants may have more negative behaviors, leading to depression, and other adverse consequences in the older adult. In the future, multi-dimensional interventions should be explored, long-term follow-up should be performed, the impact of the culture and organizational environment of nursing homes on the care behavior of nursing assistants should be assessed, and the role of technology and innovation in the optimization of care behavior (such as virtual reality training) should be elucidated to comprehensively improve the quality of aged care services.

## 4 Discussion

This study examined the key elements and formation mechanisms of quality geriatric care behavior for nursing assistants. The COM-B model indicated how nursing assistants engaged in quality care behavior. The data integration revealed four domains for the quality geriatric care behavior framework: (1) the forming causes, (2) the forming elements, (3) the forming stage, and (4) the consequences. Compared with previous literature, this study furnished novel evidence on quality care behaviors, providing rational thinking to managers of old-age care facilities.

The capacities, opportunities, and motivation of nursing assistants are crucial factors that affect the successful implementation of quality geriatric behaviors. The COM-B model is currently being used widely in the field of patient behavioral changes (37–40); however, it has not been extensively used for understanding the care behavior of nursing assistants. Improving the knowledge and skills of nursing assistants through regular professional training can enhance their self-efficacy in delivering quality care. Furthermore, it is necessary to promote a positive work perception for nursing assistants, and the management of long-term care facilities should focus on assessing and improving the level of career adaptability of nursing assistants (41). Moreover, cognitive bias can significantly impact the beliefs and attitudes of nursing assistants towards older people, potentially affecting the provided care (42). Nursing staff should be trained using the aging and disability simulation virtual reality technology to provide the experience of the older adult’s sense of action perception, improve implicit cognition towards residents, and achieve real perspective-taking. The facilities should pay attention to the organizational environment, which impacts nursing assistants’ care behavior. The institutional care climate can be improved via care-improving competitions and seminars on aging knowledge. In addition, the facilities should conduct activities to optimize the relationships among staff and leaders to promote their organizational identity and belongingness. For heavy work pressures, reasonable work tasks should be arranged, and team support should be offered (43). The nursing assistants require stable income and jobs; therefore, relatedness and growth needs should be fulfilled to realize self-value (44). Thus, it is significantly important to establish a reasonable compensation and incentive mechanism. The nursing assistants deserve additional rewards or promotion opportunities based on their work quality and professional skill qualification certificates. Studies have shown that obtaining financial and career rewards based on work quality can improve job satisfaction and increase intrinsic motivation (45, 46).

In this study, the components of quality geriatric care behavior included holistic care, personalized care, respect for the older adult, positive interaction, and empowerment. McCormack’s person-centered nursing framework emphasizes working with the patient’s beliefs and values, engagement, shared decision-making, having a sympathetic presence, and providing holistic care (47). Furthermore, other studies also promote the concept of person-oriented care. This study emphasizes on the importance of positive interaction, possibly because the family is very important in Chinese culture. Nursing assistants play the role of family members when taking care of the older adult and providing them with warmth and comfort. Positive interaction can build trust and emotional connection between nursing assistants and the older adult and compensate for the loneliness caused by the distance from relatives (11). Personalized care also emphasizes the need to understand the requirements of each older adult person and provide tailored help, which also coincides with Swanson’s theory (48), suggesting that the physical and mental conditions of the older adult living in the nursing home are more fragile; therefore, they require more care. However, at present, the level of humanistic care in nursing homes in China is relatively weak, and the shortage of professional talents restricts the quality of older adult care and the practice of humanistic care. Therefore, humanistic care practice should be included in the performance index evaluation.

The formation processes of quality geriatric care behavior consist of the negative behavior cognition stage, the practice exchange run-in stage, and the positive behavior reinforcement stage. The negative behavior cognition stage is the initial stage, where the nursing assistants compare their behavior with that of other caregivers through observation, or their negative behaviors are pointed out by other nursing assistants or managers. After an attribution analysis of the specific problem, the parties shift to the second stage. The practice exchange run-in stage is the transitional stage, which mainly focuses on how nursing assistants change their care behavior and transform it into quality care behavior for the older adult. During this period, nursing assistants may be negative and resistant to change, or they may take the initiative to change their behavior through positive teamwork. The positive behavior reinforcement stage predominantly focuses on how to maintain the changed quality of geriatric care behavior. By establishing appropriate reward and punishment mechanisms, managers can encourage nursing assistants to pursue excellence in care behavior. Currently, there is no literature on the process of quality geriatric care behavior of nursing assistants in nursing homes. However, similar processes as the Plan-Do-Study-Act (PDSA) cycle (49), a tool for improving change, exist. The process is based on the identification of the problem, altering the issue, and then sustaining the change. The process and characteristics of behavior change can provide a guide for later empirical intervention.

## 5 Limitations

This study has several limitations. First, the sample was confined to only 6 cities in 3 provinces, which are only representative of central China, which may reduce the generalizability of the acquired results. Additionally, the interviewees were selected with the help of managers and not sampled randomly; the managers might have suggested nursing assistants who were more active in their daily work and older adult people with mild personalities and better communication, which may have introduced selection bias. To ensure reliability, the studies obtained additional qualitative information from multiple stakeholder perspectives, as well as observational studies.

## 6 Conclusion

Nursing assistants are the main front-line care providers to the older adult. They are the important driving force for improving the quality of care in nursing homes, and their care behavior deserves more attention. This study employed the grounded theory approach and provided a mechanism for the formation of quality geriatric behavior in nursing assistants with Chinese cultural backgrounds. The framework indicated the consequences that influence nursing assistants’ quality care behavior in long-term care facilities. This study also elucidated the quality of geriatric care behavior, and it provided a framework for future studies on practical behavior evolution.

## Data availability statement

The raw data supporting the conclusions of this article will be made available by the authors, without undue reservation.

## Ethics statement

This research was reviewed and approved by the Ethics Committee of Tongji Medical College, Huazhong University of Science and Technology (NO. 2023-S098). The studies were conducted in accordance with the local legislation and institutional requirements. The participants provided their written informed consent to participate in this study. Written informed consent was obtained from the individuals AND/OR legal guardian/next of kin for the publication of any potentially identifiable images or data included in this article.

## Author contributions

LL: Conceptualization, Formal analysis, Investigation, Methodology, Project administration, Software, Visualization, Writing – original draft, Writing – review & editing. XB: Conceptualization, Data curation, Formal analysis, Supervision, Visualization, Writing – review & editing. XH: Formal analysis, Investigation, Methodology, Writing – original draft, Writing – review & editing. LT: Data curation, Formal analysis, Methodology, Resources, Writing – review & editing. LY: Formal analysis, Investigation, Methodology, Visualization, Writing – review & editing. HLo: Investigation, Methodology, Project administration, Validation, Writing – review & editing. SH: Investigation, Methodology, Project administration, Supervision, Writing – review & editing. XL: Investigation, Methodology, Project administration, Supervision, Writing – review & editing. YH: Conceptualization, Formal analysis, Investigation, Methodology, Resources, Validation, Writing – review & editing. XP: Formal analysis, Methodology, Resources, Supervision, Visualization, Writing – review & editing. HC: Conceptualization, Formal analysis, Project administration, Supervision, Visualization, Writing – review & editing. XY: Conceptualization, Methodology, Resources, Software, Validation, Visualization, Writing – review & editing. HLi: Data curation, Formal analysis, Funding acquisition, Methodology, Software, Validation, Writing – review & editing. SW: Formal analysis, Methodology, Project administration, Resources, Writing – review & editing. YL: Funding acquisition, Project administration, Supervision, Visualization, Writing – review & editing.

## References

[1] United Nations. World population prospects 2019: Highlights. New York: United Nations Department for Economic and Social Affairs (2019).

[2] Zhang LW Fu SJ Fang Y. Prediction of the number of and care costs for disabled elderly from 2020 to 2050: a comparison between urban and rural areas in China. Sustainability. (2020) 12:2598. doi: 10.3390/su12072598

[3] Jia LF Du YF Chu L Zhang ZJ Li FY Lyu DY. Prevalence, risk factors, and management of dementia and mild cognitive impairment in adults aged 60 years or older in China: a cross-sectional study. Lancet Public Health. (2020) 5:E661–71. doi: 10.1016/S2468-2667(20)30185-7 33271079

[4] Wei Y Zhang L. Analysis of the influencing factors on the preferences of the elderly for the combination of medical care and pension in long-term care facilities based on the Andersen model. Int J Environ Res Public Health. (2020) 17:5436. doi: 10.3390/ijerph17155436, PMID: 32731557 PMC7432067

[5] Wiederhold BK Riva G Graffigna G. Ensuring the best care for our increasing aging population: health engagement and positive technology can help patients achieve a more active role in future healthcare. Cyberpsychol Behav Soc Netw. (2013) 16:411–2. doi: 10.1089/cyber.2013.1520, PMID: 23751102

[6] Kusmaul N Bern-Klug M Bonifas R. Ethical issues in long-term care: a human rights perspective. J Hum Rights Soc Work. (2017) 2:86–97. doi: 10.1007/s41134-017-0035-2

[7] Wang Z Xing Y Yan W Sun X Zhang X Huang S. Effects of individual, family and community factors on the willingness of institutional elder care: a cross-sectional survey of the elderly in China. BMJ Open. (2020) 10:e032478. doi: 10.1136/bmjopen-2019-032478, PMID: 32075825 PMC7044895

[8] Hu J Zhang Y Wang L Shi V. An evaluation index system of basic elderly care services based on the perspective of accessibility. Int J Environ Res Public Health. (2022) 19:4256. doi: 10.3390/ijerph19074256, PMID: 35409935 PMC8998794

[9] Wu M. The relationship of pain and depression among nursing home residents: mediation factors and intervention strategies Huazhong University of Science &Technology, Wuhan (2019).

[10] Stolt R Blomqvist P Winblad U. Privatization of social services: quality differences in Swedish elderly care. Soc Sci Med. (2011) 72:560–7. doi: 10.1016/j.socscimed.2010.11.012, PMID: 21167627

[11] Lung CC Liu JYW. How the perspectives of nursing assistants and frail elderly residents on their daily interaction in nursing homes affect their interaction: a qualitative study. BMC Geriatr. (2016) 16:13. doi: 10.1186/s12877-016-0186-5, PMID: 26767789 PMC4712517

[12] Yon Y Ramiro-Gonzalez M Mikton CR Huber M Sethi D. The prevalence of elder abuse in institutional settings: a systematic review and meta-analysis. Eur J Pub Health. (2019) 29:58–67. doi: 10.1093/eurpub/cky093, PMID: 29878101 PMC6359898

[13] Liao L Feng H Jiao J Zhao Y Ning H. Correction to: nursing assistants' knowledge, attitudes and training needs regarding urinary incontinence in nursing homes: a mixed-methods study. BMC Geriatr. (2023) 23:460. doi: 10.1186/s12877-023-04108-5, PMID: 37501107 PMC10375726

[14] Sunzi K Li Y Lei C Zhou X. How do the older adults in nursing homes live with dignity? A protocol for a meta-synthesis of qualitative research. BMJ Open. (2023) 13:e067223. doi: 10.1136/bmjopen-2022-067223, PMID: 37185199 PMC10151859

[15] Cooper C Marston L Barber J Livingston D Rapaport P Higgs P. Do care homes deliver person-centred care? A cross-sectional survey of staff-reported abusive and positive behaviours towards residents from the MARQUE (managing agitation and raising quality of life) English national care home survey. PLoS One. (2018) 13:e0193399. doi: 10.1371/journal.pone.0193399, PMID: 29561867 PMC5862450

[16] Chen R Dai F Yang H Zhang X Zhang H Liu M. Caring behaviours perceived by elderly residents and their associated factors in nursing homes in Zhengzhou City of China: a qualitative study. Health Soc Care Community. (2021) 29:e126–34. doi: 10.1111/hsc.13252, PMID: 33278313

[17] Braun JA Capezuti EA. The legal and medical aspects of physical restraints and bed Siderails and their relationship to falls and fall-related injuries in nursing homes. DePaul J Health Care Law. (2000) 4:1–72.

[18] Barba BE Hu J Efird J. Quality geriatric care as perceived by nurses in long-term and acute care settings. J Clin Nurs. (2012) 21:833–40. doi: 10.1111/j.1365-2702.2011.03781.x, PMID: 21910775

[19] Murphy K. Nurses’ perceptions of quality and the factors that affect quality care for older people living in long-term care settings in Ireland. J Clin Nurs. (2007) 16:873–84. doi: 10.1111/j.1365-2702.2006.01633.x, PMID: 17462037

[20] López J Pérez-Rojo G Noriega C Velasco C. Personal and work-related factors associated with good Care for Institutionalized Older Adults. Int J Environ Res Public Health. (2021) 18:820. doi: 10.3390/ijerph18020820, PMID: 33477928 PMC7833360

[21] Castle NG. Measuring caregiver retention in nursing homes. The Gerontologist. (2021) 61:e118–28. doi: 10.1093/geront/gnab012, PMID: 33524130

[22] Gerridzen IJ Hertogh CM Joling KJ Veenhuizen RB Verschuur EM Janssen T. Caregivers' perspectives on good care for nursing home residents with Korsakoff syndrome. Nurs Ethics. (2021) 28:358–71. doi: 10.1177/0969733020921507, PMID: 32436458 PMC8151566

[23] Shin JH Hyun TK. Nurse staffing and quality of Care of Nursing Home Residents in Korea. J Nurs Scholarsh. (2015) 47:555–64. doi: 10.1111/jnu.12166, PMID: 26467903

[24] Lee A Brude S Berry MD. Long-term care: facility quality and safety. Issue Brief Health Policy Track Serv. (2018) 2018:1–48. doi: 10.1007/978-1-4020-5614-7_2010 PMID: 30681809

[25] Barnsteiner JH. Using grounded theory in nursing. J Adv Nurs. (2002) 40:370–11. doi: 10.1046/j.1365-2648.2002.24041.x

[26] Charmaz KC. Constructing grounded theory: a practical guide through qualitative analysis. Int J Qual Stud Health Well Being. (2006) 1:188–192. doi: 10.3402/qhw.v1i3.4932

[27] Oliver C. The relationship between symbolic interactionism and interpretive description. Qual Health Res. (2012) 22:409–15. doi: 10.1177/1049732311421177 21876207

[28] Strauss A Corbin J. Basics of qualitative research:Grounded theory procedures and techniques. Newbury Park, CA: Sage Publications (1990).

[29] Barrett D. Research spotlight: constructivist grounded theory. Evid Based Nurs. (2023) 26:89–90. doi: 10.1136/ebnurs-2023-103747, PMID: 37045559

[30] Charmaz K. Grounded theory methods in social justice research. Strateg Qual Inq. (2011) 4:359–80.

[31] Peters H. A-methodological saturation: a grounded theory analysis. The Counseling psychologist. (2023) 51:933–969.

[32] Saunders B Sim J Kingstone T Baker S Waterfield J Bartlam B. Saturation in qualitative research: exploring its conceptualization and operationalization. Qual Quant. (2018) 52:1893–907. doi: 10.1007/s11135-017-0574-8, PMID: 29937585 PMC5993836

[33] Glaser B Strauss A. Discovery of grounded theory: strategies for qualitative research Routledge (2017).

[34] Charmaz K. Grounded theory: objectivist and constructivist methods. Handbook of qualitative research, Sage, Thousand Oaks, CA (2000) 2:509–535.

[35] Guba EG. Criteria for assessing the trustworthiness of naturalistic inquiry. Educ Technol Res Dev. (1981) 29:75–91.

[36] Michie S van Stralen MM West R. The behaviour change wheel: a new method for characterising and designing behaviour change interventions. Implement Sci. (2011) 6:42. doi: 10.1186/1748-5908-6-42, PMID: 21513547 PMC3096582

[37] Bhandari B Narasimhan P Vaidya A Subedi M Jayasuriya R. Barriers and facilitators for treatment and control of high blood pressure among hypertensive patients in Kathmandu, Nepal: a qualitative study informed by COM-B model of behavior change. BMC Public Health. (2021) 21:1524. doi: 10.1186/s12889-021-11548-4, PMID: 34372808 PMC8351340

[38] O'Donovan B Mooney T Rimmer B Fitzpatrick P Flannelly G Doherty L. Advancing understanding of influences on cervical screening (non)-participation among younger and older women: a qualitative study using the theoretical domains framework and the COM-B model. Health Expect. (2021) 24:2023–35. doi: 10.1111/hex.13346, PMID: 34476875 PMC8628586

[39] Zhang MY Guo LN Namassevayam G Wei M Xie YY Guo YL. Factors associated with health behaviours among stroke survivors: a mixed-methods study using COM-B model. J Clin Nurs. (2024) 33:2138–52. doi: 10.1111/jocn.17103, PMID: 38590015

[40] Petersen HV Sivertsen DM Jorgensen LM Petersen J Kirk JW. From expected to actual barriers and facilitators when implementing a new screening tool: a qualitative study applying the theoretical domains framework. J Clin Nurs. (2023) 32:2867–79. doi: 10.1111/jocn.16410, PMID: 35739640

[41] Sun C Xing Y Wen Y Wan X Ding Y Cui Y. Association between career adaptability and turnover intention among nursing assistants: the mediating role of psychological capital. BMC Nurs. (2023) 22:29. doi: 10.1186/s12912-023-01187-y, PMID: 36732804 PMC9894670

[42] Thomas AK Smith AM Mazerolle M. The unexpected relationship between retrieval demands and memory performance when older adults are faced with age-related stereotypes. J Gerontol B Psychol Sci Soc Sci. (2020) 75:241–50. doi: 10.1093/geronb/gby031, PMID: 29608776

[43] Backman A Sjögren K Lövheim H Edvardsson D. Job strain in nursing homes-exploring the impact of leadership. J Clin Nurs. (2018) 27:1552–60. doi: 10.1111/jocn.14180 29148598

[44] Tuckett A Hegney D Parker D Eley RM Dickie R. The top eight issues Queensland Australia's aged-care nurses and assistants-in-nursing worried about outside their workplace: a qualitative snapshot. Int J Nurs Pract. (2011) 17:444–54. doi: 10.1111/j.1440-172X.2011.01966.x, PMID: 21939475

[45] Ikeda-Sonoda S Ichihara N Okochi J Takahashi A Miyata H. Association of care workers' job satisfaction and global happiness with change of functional performance of severely disabled elderly residents in nursing homes: a cohort and questionnaire study in Japan. BMJ Open. (2020) 10:e033937. doi: 10.1136/bmjopen-2019-033937, PMID: 33020074 PMC7537441

[46] Eisenberger R Cameron J. Detrimental effects of reward. reality or myth? Am Psychol. (1996) 51:1153–66. doi: 10.1037/0003-066X.51.11.1153 8937264

[47] McCormack B McCance T. Person-centred nursing: theory, models and methods. Blackwell Publishing: Oxford (2010).

[48] Swanson KM. Nursing as informed caring for the well-being of others. Image J Nurs Sch. (1993) 25:352–7. doi: 10.1111/j.1547-5069.1993.tb00271.x 8288305

[49] Berwick DM. Developing and testing changes in delivery of care. Ann Intern Med. (1998) 128:651–6. doi: 10.7326/0003-4819-128-8-199804150-00009 9537939

