# Supplementary material for: Identification of formation mechanism and key elements of quality geriatric care behavior of nursing assistants in nursing homes: a grounded theory study
Source: Front Public Health. 2024 Jun 27;12:1425883. doi: 10.3389/fpubh.2024.1425883 (PMC11238187; doi:10.3389/fpubh.2024.1425883)
Supplement: Supplementary file 1 [file Data_Sheet_1.docx]

**Critical Appraisal Skills Programme (CASP) qualitative checklist**

| **Guideline** | **Comments** |
| --- | --- |
| 1. Was there a clear statement of the aims of the research? | **Introduction –**Lines 115-121 |
| 2. Is a qualitative methodology appropriate? | **Methods –**Lines 124-133 |
| 3. Was the research design appropriate to address the aims of the research? | **Methods –**Lines 134-139 |
| 4. Was the recruitment strategy appropriate to the aims of the research? | **Methods –**Lines 141-150 |
| 5. Was the data collected in a way that addressed the research issue? | **Methods –**Lines 181-210 |
| 6. Has the relationship between researcher and participants been adequately considered? | **Methods –**Lines 241-244 |
| 7. Have ethical issues been taken into consideration? | **Methods –**Lines 237-239 |
| 8. Was the data analysis sufficiently rigorous? | **Methods –**Lines 211-236 |
| 9. Is there a clear statement of findings? | **Results –**Lines 245-435 |
| 10. How valuable is the research? | **Relevance to clinical practice–**Lines 536-543 |
