# Supplementary material for: Identification of formation mechanism and key elements of quality geriatric care behavior of nursing assistants in nursing homes: a grounded theory study
Source: Front Public Health. 2024 Jun 27;12:1425883. doi: 10.3389/fpubh.2024.1425883 (PMC11238187; doi:10.3389/fpubh.2024.1425883)
Supplement: Supplementary file 2 [file Data_Sheet_2.docx]

**The example outcomes of participatory observation**

| **Observation description** | **Analysis and reflection** |
| --- | --- |
| Wednesday, June 28, 2023, 14:30-17:30, XX Nursing Home  **Overview:** XX facility is a five-star nursing institution combining medical care and nursing care. The nursing assistants in the institution are licensed to work and have strict pre-job training.  **Impressive two events:** At three o 'clock in the afternoon, music was played in the hall on the first floor, and the elderly and nursing assistants danced in the hall, some elderly people danced with the elderly, some nursing assistants danced with the elderly, and everyone was brimming with happy smiles. In addition, in the outer circle of the dance floor, there are also nursing assistants pushing some elderly people who are unable to move in wheelchairs, communicating with the elderly while watching. The elderly people in wheelchairs are also very happy to take videos with their mobile phones.  Another event is that when a nursing assistants was scrubbing a bedridden old woman, although the old woman was completely unable to move (and with severe dementia), the nursing assistant still pulled the curtain of the bed, and during the operation, we can still hear her constantly communicating with the old people, saying that the weather is like today, saying that you (the grandmother) will be more comfortable after cleaning and so on. The old woman also made a sound from time to time, as if she were talking to the nursing assistant. | In the previous interview, some nursing assistants mentioned that good care is not only to meet the basic survival needs of the elderly, but also to make them live happily.  Therefore, we should interact with the elderly more, encourage the elderly can develop their own interests and hobbies, and be full of hope for life. But does it also have to do with the level of emphasis of the organization's managers, whether the manpower is sufficient, whether the managers organize these recreational activities on a regular basis?  Although the observers were all female and the elderly woman was a bedridden patient with severe dementia, the nursing assistant still closed the bed curtain to protect the privacy of the elderly woman, and in the process of operation, they continued to communicate with her, which was very humane. |
